# Association between oral function and physical pre-frailty in community-dwelling older people: a cross-sectional study

**DOI:** 10.1186/s12877-022-03409-5

**Published:** 2022-09-02

**Authors:** Asuka Tani, Shinsuke Mizutani, Saori Oku, Harukaze Yatsugi, Tianshu Chu, Xin Liu, Kiyomi Iyota, Hiro Kishimoto, Haruhiko Kashiwazaki

**Affiliations:** 1 Section of Geriatric Dentistry and Perioperative Medicine in Dentistry, Division of Maxillofacial Diagnostic and Surgical Sciences, Faculty of Dental Science, Kyushu University, 3-1-1 Maidashi, Higashi-ku, Fukuoka, 812-8582 Japan; 2 Faculty of Dental Science, Oral Health/Brain Health/Total Health Research Center, Kyushu University, 3-1-1 Maidashi, Higashi-ku, Fukuoka, 812-8582 Japan; 3 Department of Behavior and Health Sciences, Graduate School of Human-Environment Studies, Kyushu University, 744 Motooka Nishi-ku, Fukuoka City, Fukuoka, 819-0395 Japan; 4 Faculty of Arts and Science, Kyushu University, 744 Motooka Nishi-ku, Fukuoka City, Fukuoka, 819-0395 Japan

**Keywords:** Frailty, Oral function, Older people, Social activity, Muscle weakness

## Abstract

**Background:**

Few studies have examined the relationship between oral functions and the physical pre-frailty status, classified using physical function tests. This cross-sectional study aimed to clarify this association among community-dwelling older people from the Itoshima Frail Study in Itoshima Fukuoka Prefecture.

**Methods:**

Of the 1,555 individuals invited to join the study, 381 (188 males and 193 females) enrolled. Their physical pre-frailty was assessed with a classification system consisting of two physical indicators (fatigue and unintentional weight loss, determined with a questionnaire), two functional components (declined walking speed and muscle weakness, determined using a body function measuring instrument), and declined physical activity (examined using a triaxial accelerometer). Subsequently, the individuals were classified into three groups: robust, pre-frailty, and frailty. Along with the number of teeth remaining, oral functions, such as masticatory performance, tongue pressure strength, and oral diadochokinesis (ODK), were examined. Data regarding social activity and exercise habits were collected, and the individuals’ body compositions were measured. Odds ratios (ORs) and 95% confidence intervals (CIs) for the physical pre-frailty were calculated using logistic regression models.

**Results:**

In this study, 126 (33%) participants presented with physical pre-frailty. The participants in the robust group were younger, had stronger maximum handgrip strength, and walked faster than those in the physical pre-frailty group (*p* < 0.001). The robust group presented with better oral functions (masticatory performance, *p* = 0.015; oral ODK /ta/, *p* = 0.004). The physical pre-frailty status was significantly associated with age (OR, 1.111; 95% CI, 1.048–1.178; *p* < 0.001), masticatory performance (OR, 0.819; 95% CI, 0.680–0.986; *p* = 0.035), low ODK/ta/ (OR, 1.864; 95% CI, 1.069–3.250; *p* = 0.028), and low social activity (OR, 2.273; 95% CI, 1.308–3.951; *p* = 0.004).

**Conclusion:**

This study indicated that older people with higher age, lower anterior tongue movement, lower masticatory performance, and lower social activity are positively associated with physical pre-frailty.

## Background

Globally, the aging population is a common issue. Many older people need nursing care for approximately 10 years of their lives [1]. The long-term care burden for the aged has resulted in a major shortage of caregivers and funding for long-term care insurance and other services in major countries worldwide [2, 3]. Frailty and pre-frailty are symptoms of aging characterized by functional decline and vulnerability due to stressors because of reduced physiological reserve. This condition led to an increased risk of requirement for nursing care [4, 5]. The pre-frailty condition precedes frailty and is barely noticeable. There are so few signs that even frailty that changes ones’s pre-frailty physical condition is barely noticeable [6]. Several studies suggest that focusing on frailty and pre-frailty is important for preventing nursing care and concluded that exercise and nutrition interventions are among the most successful methods to reduce frailty [7, 8]. However, these reviews did not focus on oral functions, although oral functions are closely related to physical functions [9, 10] and nutrition [11].

However, some recent studies have reported that decreased oral functions, such as tongue strength and masticatory performance, are associated with frailty and pre-frailty [12, 13]. Although Watanabe et al. [14] and Shimazaki et al. [15] reported that frailty was associated with a decline in the masticatory performance and swallowing functions, respectively, they classified the frailty status using questionnaires, not subjective physical function tests. Only a few studies have investigated the relationship between oral function and physical frailty, classified using physical function tests [16, 17]. The use of questionnaire survey is simple, and it can even be performed by non-professionals; hence, it is suitable for use as a screening tool in large-scale surveys [18]. However, objective evaluations, such as physical function tests, are important because physical pre-frailty is difficult to notice independently.

To the best of our knowledge, there have been few studies focused on oral functions in older people with physical pre-frailty classified by physical function tests. We hypothesized that oral functions are associated with physical pre-frailty, which was evaluated using physical function tests. Thus, this study aimed to explore the potential association between oral function and physical pre-frailty in community-dwelling older people.

## Methods

### Participants

**Fig. 1:**
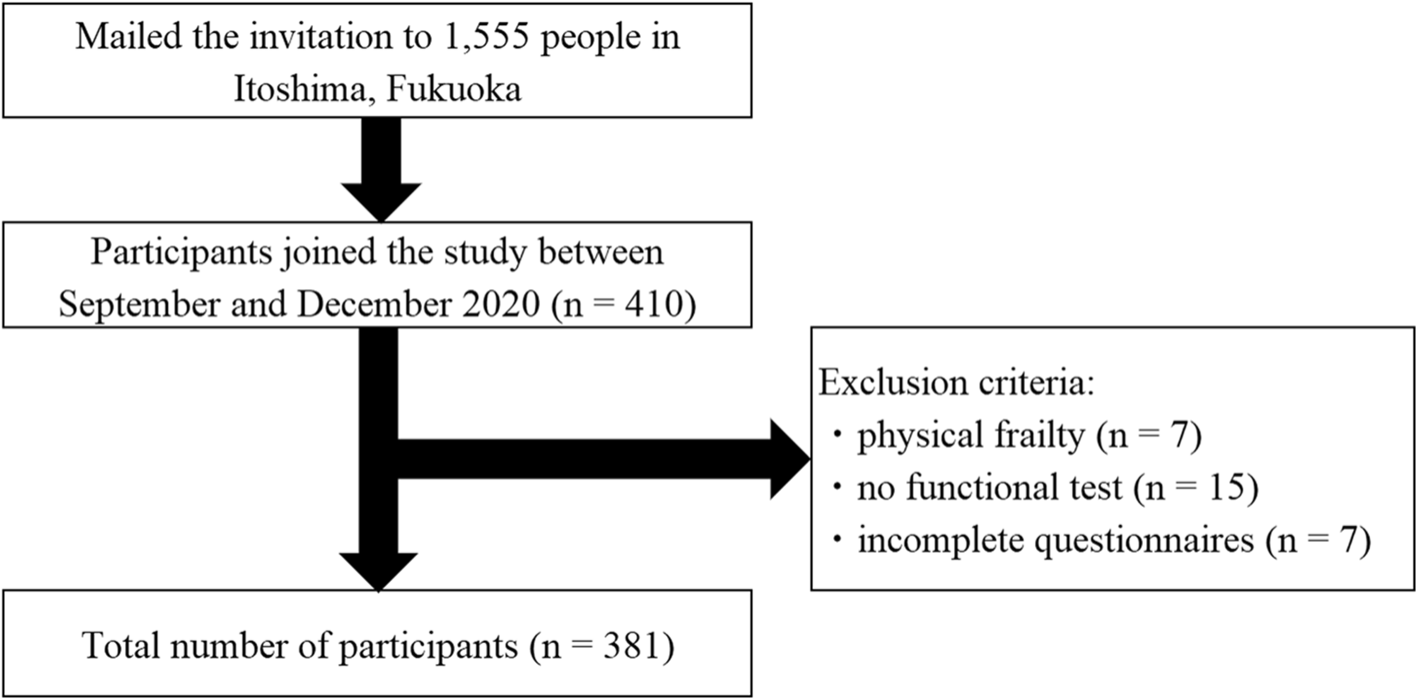
Flowchart of the participants included in the study

Invitations to enroll in this cross-sectional study were sent to 1,555 community-dwelling older people from the Itoshima Frail Study (IFS; 2017) at Itoshima City, Fukuoka Prefecture. Among them, 410 individuals participated in the study between September and December 2020; however, 29 were excluded, and the total number of participants enrolled was 381 (188 males and 193 females). These participants were classified into the robust and pre-frailty groups using the Japanese version of the frailty criteria proposed by Chen [18]. The classification consists of two functional components (muscle weakness and declined walking speed), two physical indicators (fatigue and unintentional weight loss), and low energy expenditure in terms of physical activity. These test items consist of three measurements and two questions. Each measurement and question was equally counted as 1 point, and the participants were given a score ranging from 0 to 5 points. Those with a score of 0 were included in the robust group, those with a score of 1 or 2 in the physical pre-frailty group, and those with a score of ≥ 3 in the physical frailty group. The exclusion criteria for the study were as follows: those who were frailty (*n* = 7); could not complete the oral and physical function tests because of physical disabilities (*n* = 15); and had missing data in the questionnaires about their social activities and exercise habits (*n* = 7; as shown in Fig. 1).

All participants were informed of the study and provided consent to participate. Furthermore, the participants were informed that the data obtained would not be used for other purposes or published and would not lead to any personal identification. They were free to withdraw from the study at any time. The Institutional Review Board of Kyushu University, Japan, approved this study (Application number: 202005).

### Physical function

The participants were classified into robust and pre-frailty groups using the Japanese version of the frailty criteria proposed by Chen [18]. The classification consists of two functional components (muscle weakness and declined walking speed), two physical indicators (fatigue and unintentional weight loss), and low energy expenditure in terms of physical activity. Table 1 shows the cutoff values of the measurements used in this study. Each measurement and question was equally counted as 1 point, and the participants were given a score ranging from 0 to 5 points. Those who scored 0 were included in the robust group, whereas those who scored 1 or 2 were included in the physical pre-frailty group.

**Table 1:** Definitions of each measurement for physical pre-frailty

		Definitions
Grip strength	Male	≤ 27.60 kg for BMI < 18.5, ≤ 32.10 kg for 18.5 ≤ BMI < 25, ≤ 30.35 kg for 25 ≤ BMI < 30, ≤ 29.70 kg for BMI ≥ 30
	Female	≤ 20.00 kg for BMI < 18.5, ≤ 19.40 kg for 18.5 ≤ BMI < 25, ≤ 20.40 kg for 25 ≤ BMI < 30, ≤ 20.00 kg for BMI ≥ 30
Slowness	Male	Time ≥ 3.08 s for height < 165.0 cm or time ≥ 2.84 s for height ≥ 165.0 cm
	Female	Time ≥ 3.17 s for height < 152.3 cm or time ≥ 2.97 s for height ≥ 152.3 cm
Exhaustion		A positive answer to either of 2 self-reported questions. Participants were asked how they felt in last 1 month: “Do you feel that everything you do is an effort?;” “Do you feel exhausted without any reason?”
Weakness	Male	≤ 27.60 kg for BMI < 18.5, ≤ 32.10 kg for 18.5 ≤ BMI < 25, ≤ 30.35 kg for 25 ≤ BMI < 30, ≤ 29.70 kg for BMI ≥ 30
	Female	≤ 20.00 kg for BMI < 18.5, ≤ 19.40 kg for 18.5 ≤ BMI < 25, ≤ 20.40 kg for 25 ≤ BMI < 30, ≤ 20.00 kg for BMI ≥ 30
Shrinking		Unintentional weight loss of > 2–3 kg in the past 6 months
Low physical activity	Male	≤ 6.63 kcal/kg/d
	Female	≤ 8.24 kcal/kg/d

*BMI* Body mass index

#### Grip strength

Grip strength, used as an indicator of muscle weakness, was measured using an electronic grip strength meter (Grip D TKK-5401, Takei Scientific Instruments Co. Ltd., Niigata, Japan). The second joint of the index finger was at a right angle to the grip meter, and the display was on the outside. The subjects were instructed to keep their legs apart, lower their arms naturally, and grip the meter completely [19]. The measurements were repeated twice, alternating between hands. Representative values for both hands were adopted as the indicator of weakness. The cutoff values considering sex and age were obtained from a previous study [19].

#### Slowness

A five-meter maximum gait speed was taken as an indicator of declined walking speed. The participants were instructed to walk for 11 m at the maximum walking speed possible without running. The measurements were carried out twice, and the time was recorded between the third and eighth meters. The maximum value obtained was adopted as the indicator of declined walking speed. The cutoff values considering sex and age were obtained from a previous study [19].

#### Exhaustion

The participants were asked the following questions to determine the level of fatigue over the past 1 month: “Did you feel that everything you did was an effort?” and “Did you feel exhausted without any reason?” [19].

#### Shrinking

The participants were asked whether they experienced any unintended weight loss of > 2–3 kg in the last 6 months [19].

#### Low physical activity

The physical activity of participants was objectively measured using a triaxial accelerometer (Active Style Pro, HJA350-IT, Omron Healthcare, Co Ltd, Kyoto, Japan). The participants wore this device on the waist or hips for at least 1 week. Low physical activity was defined as scores within the lowest 20% of daily energy expenditure for physical activity, stratified by sex. Data were quantified as kilocalories per kilogram of body weight spent per day (kcal/kg/day). The determination of the duration of the fit was based on the longest period of generally recommended time. We defined the valid day as that of > 600 min, and the eligibility criterion for the participants was those with ≥ 3 valid days [19].

### Body composition

In addition to the height and weight, the body fat and skeletal muscle masses of the trunk were measured using a commercial multi-frequency body composition instrument (MC-980A plus, TANITA Co., Tokyo, Japan). Subsequently, the body mass index (BMI) was calculated using the values for weight and height.

### Oral function

Four trained dentists performed the oral examinations after the calibration. Moreover, they developed a measurement protocol before the initiation of the investigation, and measurements were conducted according to the procedure of the protocol. They examined and counted the number of teeth present in each participant, and teeth with increased mobility (Miller’s classification 3) and those without a crown were excluded from the count as they were nonfunctional [20, 21].

#### Masticatory performance

A test chewing gum (Masticatory Performance Evaluating Gum XYLITOL; Lotte Co Ltd.; 70 × 20 × 1 mm; 30 g) containing a dyed substance that changes color from green to red when mixed with saliva was used for the test. This test chewing gum was developed to provide a quick and objective evaluation in various situations as well as to assess the masticatory performance in older people [22–25]. The participants were instructed to chew the gum freely at a rate of once per second for 60 s at a comfortable point. The color of the chewed gum was measured on a 10-point scale immediately after chewing to minimize the color change with time [21].

#### Tongue pressure

Maximum tongue pressure was measured using a tongue depressor from JMS (TPM-01, JMS Co. Ltd., Hiroshima, Japan). The maximum tongue pressure was defined as the pressure applied by the tongue when participants pressed the balloon attached to the tongue depressor to the anterior part of the palate. The maximum value was recorded after three measurements. In the case of a denture user, the pressure was measured with the denture in place [26].

#### Oral Diadochokinesis (ODK)

The speed and smoothness of the tongue and lip movements were comprehensively measured to determine the ODK. The participants repeatedly pronounced the syllables /pa/, /ta/, and /ka/ in succession with 5 s for each syllable, and the pronounciations were recorded using a counter machine (Kenkokun Handy, Takei Scientific Instruments, Niigata, Japan). Simultaneously, the machine calculated the number of pronunciations per second. When the count was less than six times per second, the tongue–lip motor function was considered to be decreased [26].

### Data collection for other variables

The participants answered a questionnaire during the measurement session. The questionnaire consisted of questions about their social activity and exercise habits, in addition to sex and age. The answer was coded as a “yes” when they responded affirmatively to participate in at least one of the following activities: local events (such as volunteer groups and hobby activity groups); community associations; senior citizen clubs; and other activities [27]. The participants described their exercise habits, including the frequency of their weekly activity and time spent in each session during the previous month. The following exercise habits were included: normal or brisk walking; repeated movement exercise; ground golf; hiking; bowling; ballroom dancing; cycling; and other types of exercises. The exercise habit was defined as performing the activity at least once per week during leisure time [28].

### Sample size calculation

The sample size was estimated using G* Power (ver. 3.1.9.4, Universität Kiel, Kiel, Germany), and the minimum sample sizes for the groups were calculated. A previous study revealed that the mean ± standard deviation values for tongue pressure in the robust and frailty groups were 29.5 ± 8.2 and 24.1 ± 9.4 kPa, respectively [29]. A minimum total sample size of 88 was calculated considering an effect size of 0.61, α of 0.05, and a power (1 − β) of 0.80.

### Statistical analysis

A *P*-value of < 0.05 was considered to be statistically significant. The Statistical Package for the Social Sciences version 26.0 software program (IBM Corporation., Armonk, NY, USA) performed analyses.

#### Comparison of the robust and physical pre-frailty groups

The participants were divided into two groups according to Chen’s criteria: robust group (*n* = 188) and physical pre-frailty group (*n* = 193). Student’s *t*-test and chi-square test were used to investigate significant differences in mean values between the two groups.

#### Binary logistic regression analysis

The odds ratios (ORs) and 95% confidence intervals (CIs) were calculated using the multivariate logistic regression after simultaneous controlling. The robust and physical pre-frailty groups were used as dependent variables. In addition to age and sex, each variable with a *p*-value of < 0.05 in the *t*-test or chi-square test was selected as an independent variable. Hence, age (continuous), sex (0, male; 1, female), masticatory performance (continuous), ODK/ta/ (0, normal; 1, decreased), and social activity (0, yes; 1, no) were considered as independent variables for the binomial logistic regression analysis. Additionally, for the independent variables with a *p*-value of < 0.20 in the *t*-test or chi-square test, sensitivity analyses were performed. Hence, age, sex, BMI (continuous), body fat mass (continuous), skeletal muscle mass (continuous), number of remaining teeth (continuous), masticatory performance, tongue pressure (continuous), ODK/pa/, /ta/, /ka/ (0, normal; 1, decreased), and social activity were considered as independent variables for the binomial logistic regression analysis. Grip strength and walking speed were not considered as independent variables because they were used for the diagnosis of physical pre-frailty.

## Results

Table 2 shows the study participants’ characteristics. The study enrolled 381 individuals with a mean age of 72.6 years. The participants in the robust group were younger, presented with stronger maximum handgrip strength, and walked faster than those in the physical pre-frailty group (*p* < 0.001). Moreover, those in the robust group had a lower body fat mass of the body trunk and more skeletal muscle mass (*p* < 0.05). The robust group presented with better oral functions in terms of the masticatory performance and ODK /ta/ (*p* = 0.015 and *p* = 0.004, respectively); however, there were no significant differences in the number of remaining teeth and other oral functions. The participants in the robust group had a more social activity than those in the physical pre-frailty group (*p* = 0.005). However, there were no significant differences in exercise habits between the two groups.

**Table 2:** Comparison of variables between the robust and physical pre-frailty groups

Variable		Total (*n* = 381)	Robust group (*n* = 255)	Physical pre-frailty group (*n* = 126)	*p*-value, 95% Confidence interval
Age (years)		72.6 ± 3.9	72.1 ± 3.8	73.6 ± 4.1	< 0.001, − 2.302– − 0.597^*^
Sex (Male)		188 (49)	130 (51)	58 (46)	0.363, 0.795–1.870^†^
Body mass index (kg/m^2^)		23.0 ± 3.3	22.9 ± 3.1	23.4 ± 3.1	0.161, − 1.250–0.208^*^
	Male		23.0 ± 2.8	24.0 ± 3.4	
	Female		22.7 ± 3.4	22.8 ± 3.6	
Maximum grip strength (kg)		29.4 ± 7.9	30.7 ± 7.8	26.7 ± 7.6	< 0.001, 2.457–5.733^*^
	Male		37.4 ± 4.6	33.0 ± 5.2	
	Female		23.8 ± 2.7	21.2 ± 4.4	
5 m maximum gait speed (s)		2.7 ± 0.5	2.6 ± 0.4	2.8 ± 0.6	< 0.001, − 0.371– − 0.134^*^
Body fat mass (kg)		15.0 ± 6.1	14.9 ± 6.1	16.1 ± 6.5	0.101, − 2.509–0.225^*^
	Male		13.0 ± 5.3	15.4 ± 6.1	
	Female		17.0 ± 6.2	16.7 ± 6.9	
Skeletal muscle mass (kg)		40.6 ± 7.8	41.1 ± 7.7	39.6 ± 8.0	0.090, − 0.231–3.147^*^
	Male		47.7 ± 4.9	47.0 ± 5.0	
	Female		34.2 ± 2.8	33.3 ± 2.9	
Number of remaining teeth		22.6 ± 7.4	23.0 ± 6.9	21.7 ± 8.1	0.109, − 0.306–3.027^*^
Masticatory performance		7.9 ± 1.2	8.0 ± 1.1	7.7 ± 1.3	0.015, 0.066–0.600^*^
Tongue pressure (kPa)		36.7 ± 7.5	37.1 ± 7.3	35.8 ± 8.0	0.127, − 0.373–2.975^*^
ODK /pa/	Normal	319 (84)	220 (86)	99 (79)	0.055, 0.335–1.016^†^
	Decreased	62 (16)	35 (14)	27 (21)	
ODK /ta/	Normal	312 (82)	219 (86)	93 (74)	0.004, 0.272–0.788^†^
	Decreased	69 (18)	36 (14)	33 (26)	
ODK /ka/	Normal	236 (62)	166 (65)	70 (56)	0.071, 0.434–1.036^†^
	Decreased	145 (38)	89 (35)	56 (44)	
Social activity	Yes	306 (80)	215 (84)	91 (72)	0.005, 1.234–3.462^†^
	No	75 (20)	40 (16)	35 (28)	
Exercise habit	Yes	214 (56)	148 (58)	66 (52)	0.295, 0.819–1.931^†^
	No	167 (44)	107 (42)	60 (48)	

Values are presented as mean ± standard deviation or number (%)

*p*-values and 95% CIs of the comparison between the robust and physical pre-frailty groups were obtained using the Student’s t-test and Pearson’s chi-square test

ODK was considered to be decreased if the value was less than six times per second

After adjusting for confounding factors in the binomial logistic regression analysis, physical pre-frailty was found to be significantly associated with age (OR, 1.111; 95% CI, 1.048–1.178; *p* < 0.001), female sex (OR, 1.214; 95% CI, 0.768–1.919; *p* < 0.407), masticatory performance (OR, 0.819; 95%CI, 0.680–0.986; *p* = 0.035), low ODK/ta/ (OR, 1.864; 95%CI, 1.069–3.250, *p* = 0.028), and low social activity (OR, 2.273; 95%CI, 1.308–3.951; *p* = 0.004). The Hosmer–Lemeshow test revealed that the model had an acceptable fit with the data, with a chi-square statistical value of 10.729 (*p* = 0.218) and an accuracy of discrimination of 66.9% (Table 3). The results of the sensitivity analysis were the same as the results of the original logistic regression analysis in the final model.

**Table 3:** Adjusted odds ratios and 95% confidence intervals for physical pre-frailty

Variables		Odds ratio	95% Confidence interval	*p*-value
Age		1.111	1.048–1.178	< 0.001
Sex	Male	1.000		
	Female	1.214	0.768–1.919	0.407
Masticatory performance		0.819	0.680–0.986	0.035
ODK /ta/	Normal	1.000		
	Decreased	1.864	1.069–3.250	0.028
Social activity	Yes	1.000		
	No	2.273	1.308–3.951	0.004

Model fit (simultaneous selection): Hosmer–Lemeshow test revealed *p*-value of 0.218, and the accuracy of discrimination was 66.9%

Dependent variable: physical pre-frailty (0: robust, 1: physical pre-frailty)

Independent variables: age (continuous), sex (0: male, 1: female), masticatory performance (continuous), ODK /ta/ (0: normal, 1: decreased), and social activity (0: yes, 1: no)

ODK, oral diadochokinesis

The effect size and statistical power in the post hoc analysis were 0.25 and 0.77, respectively, when the masticatory performances between the robust and physical pre-frailty groups were compared.

## Discussion

The results of this study showed that individuals with slow anterior tongue movement and the diminished masticatory performance had a higher possibility of developing physical pre-frailty than those with normal oral functions. Pre-frailty is the critical stage wherein the individual can either return to the robust state or progress to the frailty state; hence, appropriate interventions are effective during this stage [8, 30]. A recent study reported that oral conditions, such as few remaining teeth, deteriorating oral health, and masticatory function impairment, were more frequently associated with frailty. Moreover, a previous study reported that slow anterior tongue movement was associated with prolonged chewing time [31]. Additionally, tongue pressure has been reported to be mildly associated with frailty (low quality of evidence) [32]. The current study results were similar to those reported in the aforementioned review article.

Anterior tongue movement, measured as ODK/ta/, is a part of the masticatory performance to place food on the molars and buccal muscles [33]. It plays a critical role in eating efficiently and maintaining a sufficient nutrient intake [34]. For example, an unbalanced diet consisting mainly of carbohydrates with insufficient proteins, minerals, and vitamins leads to pre-frailty. Moreover, Izuno reported that ODK /ta/ is associated with many indicators of physical function, such as bending forward in a sitting position, balance, functional reach test, grip strength, and timed up and go test [9]. Although the direct association between oral function and physical pre-frailty was not demonstrated in the current cross-sectional study, the decrease in oral function observed in the participants might precede the physical pre-frailty. Iyota et al. reported a difference in improvement in the body composition following tongue lifting training between the robust and pre-frailty/frailty groups and indicated the importance of protein intake [35]. Additional studies are required to investigate the effects of improving oral function and nutritional education in preventing physical pre-frailty.

A previous study reported that maximum isometric tongue pressure was independently related to frailty [29]; however, no such association was observed in the present study. This may be because the previous study evaluated only the tongue pressure and not the masticatory function. Moreover, that study used questionnaires on frailty, which were different from the ones used in the present study. Thus, further studies are required to examine the association between tongue pressure and physical frailty/pre-frailty in the future.

Furthermore, no statistically significant association between physical pre-frailty and skeletal muscle mass was observed in this study. Makizako reported that skeletal muscle weakness preceded the decrease in skeletal muscle mass in older people with social frailty [36]. Moreover, Basaran reported that a decrease in muscle mass was associated with frailty [37]. These studies may indicate that a skeletal muscle mass decrease is a specific symptom of frailty, not pre-frailty, and may support our results. A cohort study might be needed to investigate the changes in skeletal muscle mass in individuals with pre-frailty.

Frailty has three phenotypes: physical, mental, and social [6]. Therefore, it may be reasonable to assume that social activity is associated with physical pre-frailty in the current study. A recent prospective cohort study showed that the symptoms of physical frailty predict the development of social frailty [38]. Another cross-sectional study reported associations between oral, social, and physical frailties [16]. Oral frailty was evaluated using some oral function in the current study; therefore, it is interesting to note that the oral function was directly involved in the development of physical or social frailty.

Although several previous studies have evaluated the relationship between the oral condition and frailty or pre-frailty [33, 39], assessment tools, such as Fried’s physical frailty phenotype, the Kihon checklist, and other types of questionnaires on physical activity, were used. Therefore, individuals with pre-frailty were considered robust, and their existing state was overlooked. As a result, they must have lost the opportunity to return to the healthy state [18] In the current study, we determined the pre-frailty state using several physical function tests. The percentages of individuals in the robust, physical pre-frailty and frailty states were 66%, 32%, and 2%, respectively. These findings are similar to those in the study by Makino et al., where 49%, 47%, and 3.6% of the individuals belonged to the robust, pre-frailty, and frailty groups, respectively [40]. However, the participants in the present study were voluntarily enrolled in the survey, which might have increased the ratio of robust people. Therefore, these results must be carefully interpreted.

There were some limitations in the present study. First, we could not determine the causal relationship between pre-frailty and oral function because of the study’s cross-sectional nature. Therefore, the effect of lifestyle and oral function on the onset of pre-frailty must be assessed using cohort studies. Second, this study was conducted on older people living within a limited area. Additional studies comprising a wider range of older adults living in the community may be needed in the future. Third, the participants included in this study expressed their interest in enrolling in the survey; hence, they might have already been interested in social activities. Fourth, as we did not present the results of the inter-rater and intra-rater reliability, there might be measurement bias. Finally, many confounders are related to physical pre-frailty, such as an unbalanced diet, polypharmacy, decreased motivation, depression [41], and swallowing [42]; however, the present study did not examine all the factors.

## Conclusion

The results of this study showed that slow anterior tongue movement, which was measured as ODK/ta/, and low masticatory performance were substantially associated with physical pre-frailty among community-dwelling older people.

## Data Availability

The dataset from this study are available from the corresponding author on reasonable request.

## Abbreviations

OR: Odds ratio
CI: Confidence interval
ODK: Oral diadochokinesis
BMI: Body mass index

## References

[1] Hosokawa R, Ojima T, Myojin T, Aida J, Kondo K, Kondo N (2020). Associations between healthcare resources and healthy life expectancy: a descriptive study across secondary medical areas in Japan. Int J Environ Res Public Health.

[2] World Health Organization. World health statistics 2021. https://apps.who.int/iris/bitstream/handle/10665/342703/9789240027053-eng.pdf. Accessed 8 Nov 2021.

[3] Texas Health and Human Services Commission. A Profile of informal caregiving in Texas. December 2020. https://www.chcs.org/media/Report-2020-A-Profile-of-Informal-Caregiving-Report-TX.pdf. Accessed 7 Nov 2021.

[4] Xue QL (2011). The frailty syndrome: definition and natural history. Clin Geriatr Med.

[5] Fried LP, Tangen CM, Walston J, Newman AB, Hirsch C, Gottdiener J (2001). Frailty in older adults: evidence for a phenotype. J Gerontol A Biol Sci Med Sci.

[6] Gordon SJ, Baker N, Kidd M, Maeder A, Grimmer KA (2020). Pre-frailty factors in community-dwelling 40–75 year olds: opportunities for successful ageing. BMC Geriatr.

[7] Hoogendijk EO, Afilalo J, Ensrud KE, Kowal P, Onder G, Fried LP (2019). Frailty: implications for clinical practice and public health. Lancet.

[8] Matsushita E, Okada K, Ito Y, Satake S, Shiraishi N, Hirose T (2017). Characteristics of physical prefrailty among Japanese healthy older adults. Geriatr Gerontol Int.

[9] Izuno H, Hori K, Sawada M, Fukuda M, Hatayama C, Ito K (2016). Physical fitness and oral function in community-dwelling older people: a pilot study. Gerodontology.

[10] Slashcheva LD, Karjalahti E, Hassett LC, Smith B, Chamberlain AM (2021). A systematic review and gap analysis of frailty and oral health characteristics in older adults: a call for clinical translation. Gerodontology.

[11] van de Rijt LJM, Feast AR, Vickerstaff V, Sampson EL, Lobbezoo F (2021). Oral function and its association with nutrition and quality of life in nursing home residents with and without dementia: a cross-sectional study. Gerodontology.

[12] Watanabe Y, Yamada Y, Yoshida T, Yokoyama K, Miyake M, Yamagata E (2020). Comprehensive geriatric intervention in community-dwelling older adults: a cluster-randomized controlled trial. J Cachexia Sarcopenia Muscle.

[13] Tanaka T, Takahashi K, Hirano H, Kikutani T, Watanabe Y, Ohara Y (2018). Oral frailty as a risk factor for physical frailty and mortality in community-dwelling elderly. J Gerontol A Biol Sci Med Sci.

[14] Watanabe D, Yoshida T, Yokoyama K, Yoshinaka Y, Watanabe Y, Kikutani T (2020). Association between mixing ability of masticatory functions measured using color-changing chewing gum and frailty among Japanese older adults: the kyoto-kameoka study. Int J Environ Res Public Health.

[15] Shimazaki Y, Nonoyama T, Tsushita K, Arai H, Matsushita K, Uchibori N (2020). Oral hypofunction and its association with frailty in community-dwelling older people. Geriatr Gerontol Int.

[16] Hironaka S, Kugimiya Y, Watanabe Y, Motokawa K, Hirano H, Kawai H (2020). Association between oral, social, and physical frailty in community-dwelling older adults. Arch Gerontol Geriatr.

[17] Hihara T, Goto T, Ichikawa T (2020). Assessment of potential clinical cascade between oral hypofunction and physical frailty: Covariance structure analysis in a cross-sectional study. J Oral Rehabil.

[18] Chen S, Chen T, Kishimoto H, Susaki Y, Kumagai S (2020). Development of a fried frailty phenotype questionnaire for use in screening community-dwelling older adults. J Am Med Dir Assoc.

[19] Chen SM, Honda T, Chen T, Narazaki K, Haeuchi Y, Supartini A (2015). Screening for frailty phenotype with objectively-measured physical activity in a west Japanese suburban community: evidence from the Sasaguri Genkimon Study. BMC Geriatr.

[20] Miller PD (2018). Miller classification of marginal tissue recession revisited after 35 years. Compend Contin Educ Dent.

[21] Mizutani S, Egashira R, Yamaguchi M, Tamai K, Yoshida M, Kato T (2021). Changes in oral and cognitive functions among older Japanese dental outpatients: a 2-year follow-up study. J Oral Rehabil.

[22] Kamiyama M, Kanazawa M, Fujinami Y, Minakuchi S (2010). Validity and reliability of a Self-Implementable method to evaluate masticatory performance: use of color-changeable chewing gum and a color scale. J Prosthodont Res.

[23] Tarkowska A, Katzer L, Ahlers MO (2017). Assessment of masticatory performance by means of a color-changeable chewing gum. J Prosthodont Res.

[24] Kugimiya Y, Watanabe Y, Shirobe M, Motohashi Y, Motokawa K, Edahiro A (2021). A comparison of colorimetric and visual methods for the assessment of masticatory performance with color-changeable chewing gum in older persons. J Dent Sci.

[25] Senoo S, Iwasaki M, Kimura Y, Kakuta S, Masaki C, Wada T (2020). Combined effect of poor appetite and low masticatory function on sarcopenia in community-dwelling Japanese adults aged ≥ 75 years: A 3-year cohort study. J Oral Rehabil.

[26] Iyota K, Mizutani S, Oku S, Asao M, Futatsuki T, Inoue R (2020). A cross-sectional study of age-related changes in oral function in healthy Japanese individuals. Int J Environ Res Public Health.

[27] Yamaguchi M, Yoshida T, Yamada Y, Watanabe Y, Nanri H, Yokoyama K (2018). Sociodemographic and physical predictors of non-participation in community based physical checkup among older neighbors: a case-control study from the Kyoto-Kameoka longitudinal study. Japan BMC Public Health.

[28] Kishimoto H, Ohara T, Hata J, Ninomiya T, Yoshida D, Mukai N (2016). The long-term association between physical activity and risk of dementia in the community: the Hisayama Study. Eur J Epidemiol.

[29] Satake A, Kobayashi W, Tamura Y, Oyama T, Fukuta H, Inui A (2019). Effects of oral environment on frailty: particular relevance of tongue pressure. Clin Interv Aging.

[30] Apóstolo J, Cooke R, Bobrowicz-Campos E, Santana S, Marcucci M, Cano A (2018). Effectiveness of interventions to prevent pre-frailty and frailty progression in older adults: a systematic review. JBI Database System Rev Implement Rep..

[31] Kent RD, Kim Y, Chen LM (2022). Oral and laryngeal diadochokinesis across the life span: a scoping review of methods, reference data, and clinical applications. J Speech Lang Hear Res.

[32] Dibello V, Zupo R, Sardone R, Lozupone M, Castellana F, Dibello A (2021). Oral frailty and its determinants in older age: a systematic review. Lancet Healthy Longev.

[33] Yamada A, Kanazawa M, Komagamine Y, Minakuchi S (2015). Association between tongue and lip functions and masticatory performance in young dentate adults. J Oral Rehabil.

[34] Iwasaki M, Motokawa K, Watanabe Y, Shirobe M, Inagaki H, Edahiro A (2020). A two-year longitudinal study of the association between oral frailty and deteriorating nutritional status among community-dwelling older adults. Int J Environ Res Public Health.

[35] Iyota K, Mizutani S, Kishimoto H, Oku S, Tani A, Yatsugi H (2021). Effect of isometric tongue lifting exercise on oral function, physical function, and body composition in community-dwelling older individuals: a pilot study. Gerontology.

[36] Makizako H, Kubozono T, Kiyama R, Takenaka T, Kuwahata S, Tabira T (2019). Associations of social frailty with loss of muscle mass and muscle weakness among community-dwelling older adults. Geriatr Gerontol Int.

[37] Güngör Başaran AY, Akal YE (2021). Nutrition status, muscle mass, and frailty in older people: a cross-sectional study conducted in Cyprus. J Am Coll Nutr.

[38] Nagai K, Tamaki K, Kusunoki H, Wada Y, Tsuji S, Itoh M (2020). Physical frailty predicts the development of social frailty: a prospective cohort study. BMC Geriatr.

[39] Arai H, Satake S (2015). English translation of the Kihon Checklist. Geriatr Gerontol Int.

[40] Makino K, Lee S, Bae S, Chiba I, Harada K, Katayama O (2021). Prospective associations of physical frailty with future falls and fear of falling: a 48-month cohort study. Phys Ther..

[41] Siriwardhana DD, Hardoon S, Rait G, Weerasinghe MC, Walters KR (2018). Prevalence of frailty and prefrailty among community-dwelling older adults in low-income and middle income countries: a systematic review and meta-analysis. BMJ Open.

[42] González-Fernández M, Humbert I, Winegrad H, Cappola AR, Fried LP (2014). Dysphagia in old-old women: prevalence as determined according to self-report and the 3-ounce water swallowing test. J Am Geriatr Soc.

